# Prelamin A causes aberrant myonuclear arrangement and results in muscle fiber weakness

**DOI:** 10.1172/jci.insight.120920

**Published:** 2018-10-04

**Authors:** Yotam Levy, Jacob A. Ross, Marili Niglas, Vladimir A. Snetkov, Steven Lynham, Chen-Yu Liao, Megan J. Puckelwartz, Yueh-Mei Hsu, Elizabeth M. McNally, Manfred Alsheimer, Stephen D.R. Harridge, Stephen G. Young, Loren G. Fong, Yaiza Español, Carlos Lopez-Otin, Brian K. Kennedy, Dawn A. Lowe, Julien Ochala

**Affiliations:** 1 School of Basic and Medical Biosciences, Faculty of Life Sciences & Medicine, and; 2 Proteomics Facility, Centre of Excellence for Mass Spectrometry, King’s College London, London, United Kingdom.; 3 Buck Institute for Research on Aging, Novato, California, USA.; 4 Center for Genetic Medicine, Northwestern University, Chicago, Illinois, USA.; 5 Department of Cell and Developmental Biology, University of Würzburg, Würzburg, Germany.; 6 Department of Medicine, University of California Los Angeles, Los Angeles, California, USA.; 7 Departamento de Bioquímica y Biología Molecular, Facultad de Medicina, Instituto Universitario de Oncología (IUOPA), Universidad de Oviedo, Oviedo, Spain.; 8 Centro de Investigación Biomédica en Red de Cáncer (CIBERONC), Spain.; 9 Departments of Biochemistry and Physiology, Yong Loo Lin School of Medicine, National University of Singapore, Singapore.; 10 Centre for Healthy Ageing, National University Health System, Singapore.; 11 Singapore Institute for Clinical Sciences, Singapore.; 12 Divisions of Rehabilitation Science and Physical Therapy, Department of Rehabilitation Medicine, School of Medicine, University of Minnesota, Minneapolis, Minnesota, USA.

**Keywords:** Aging, Muscle Biology, Genetic diseases, Neuromuscular disease

## Abstract

Physiological and premature aging are frequently associated with an accumulation of prelamin A, a precursor of lamin A, in the nuclear envelope of various cell types. Here, we aimed to underpin the hitherto unknown mechanisms by which prelamin A alters myonuclear organization and muscle fiber function. By experimentally studying membrane-permeabilized myofibers from various transgenic mouse lines, our results indicate that, in the presence of prelamin A, the abundance of nuclei and myosin content is markedly reduced within muscle fibers. This leads to a concept by which the remaining myonuclei are very distant from each other and are pushed to function beyond their maximum cytoplasmic capacity, ultimately inducing muscle fiber weakness.

## Abstract 2

Following prelamin A accumulation in myofibers, the abundance of nuclei and myosin content within muscle fibers is reduced, ultimately inducing muscle fiber weakness.

## Graphical abstract

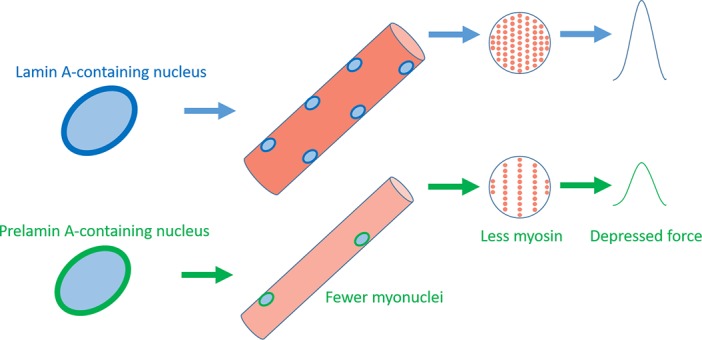

## Introduction

Biological aging involves complex dysfunctional cellular processes with unclear underlying mechanisms, including a potential involvement of alterations at the nuclear level in a wide range of tissues (1). Normal nuclear function requires lamin A, a protein located at the inner nuclear envelope, where it regulates nuclear integrity, architecture, and chromatin organization (2). Defective processing of lamin A and accumulation of its precursors, progerin and/or prelamin A, occurs during physiological aging (3, 4) and is also responsible for premature aging syndromes (2, 5). Symptoms include growth impairment, bone and skin abnormalities, joint contractures, and muscle dysfunction. In the present study, we aimed to determine whether and how high levels of prelamin A deteriorate the function of skeletal muscle fibers.

Myofibers contain several hundred peripherally located nuclei. Each of them controls protein synthesis in a defined volume of cytoplasm termed the myonuclear domain (MND). Regular positioning of these nuclei is essential for optimal nuclear cooperation, MND size, and efficient regulation and distribution of gene products (6). Here, we tested the hypothesis that an accumulation of prelamin A would alter nuclear number and positioning, ultimately disrupting the ability of fibers to generate force. To test this, we used various transgenic mouse models that mimic premature aging syndromes, wherein the composition of nuclear envelope proteins is altered. We isolated and membrane-permeabilized individual muscle fibers, then ran a series of contractile and morphological analyses, including an evaluation of the 3D organization of nuclei, using our image analysis algorithm applied to reconstructed confocal images (7).

## Results

**Figure 1:**
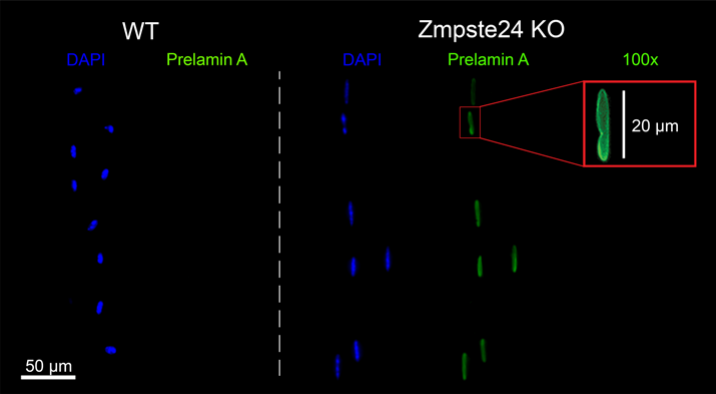
Prelamin A accumulation in Zmpste24-deficient myonuclei. Typical EDL myofibers immunostained for nuclei (blue) and prelamin A (green). WT refers to 4-month-old WT mice, while Zmpste24 KO refers to age-matched homozygous Zmpste24-deficient mice. Images were taken using a ×20 objective, apart from the inset (×100 objective). Note that all the nuclei from Zmpste24 KO mice were prelamin A positive, while none of the WT nuclei exhibited prelamin A.

Zmpste24-deficient mice represent a valuable animal model of premature/disease-induced aging, as they lack zinc metallopeptidase STE24 (Zmpste24) enzyme (8–10), which is responsible for the processing of prelamin A. As a consequence, these animals accumulate unprocessed prelamin A in their nuclei, mimicking human progeria syndromes (Figure 1).

### Myofibers lacking Zmpste24 have impaired nuclear number, MND size, and protein content.

**Figure 2:**
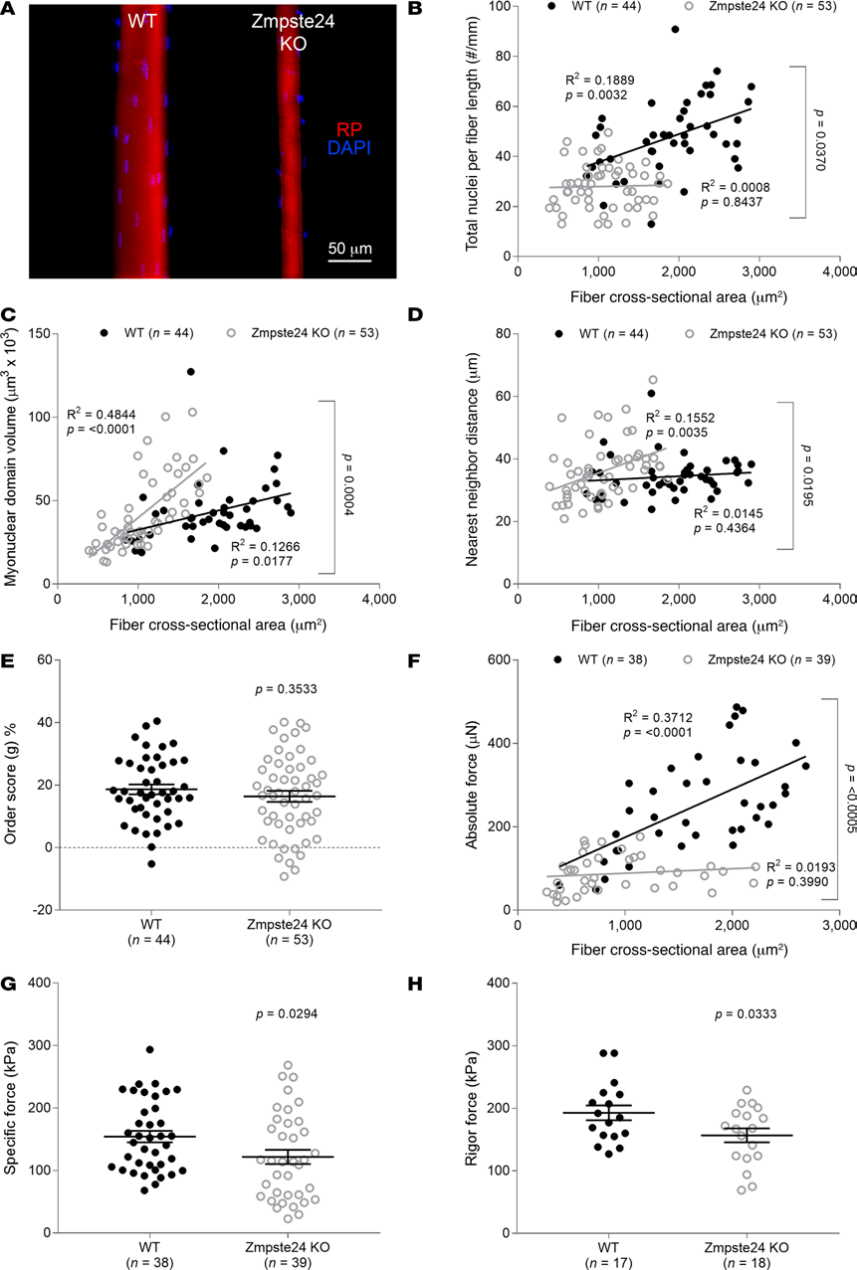
Altered myonuclear organization and force production in the absence of Zmpste24. (**A**) Typical confocal images (×20 objective) of myofibers from 4-month-old WT (*n* = 5 mice, *n* = 44 fibers) and homozygous Zmpste24-deficient (Zmpste24 KO, *n* = 5 mice, *n* = 53 fibers) mice. These fibers were stained for nuclei (DAPI, blue) and actin (rhodamine phalloidin [RP], red). In **B**–**H**, data are presented as mean ± SEM, and as scatter plots wherein individual points correspond to single muscle fibers. Statistical tests included normality tests, *t* tests, and Pearson’s product moment correlation (to evaluate linear relationships).

Muscle fibers and nuclei are commonly analyzed using muscle cross sections. This methodological approach has numerous limitations and does not allow accurate enumeration of nuclei or their distribution within fibers, as only 2 dimensions are visualized unless multiple sections are studied. To address this, we isolated 97 individual myofibers and analyzed confocal reconstructions in 3D (Figure 2A). Our data showed that the overall number of nuclei per millimeter fiber length was significantly lower in mice lacking Zmpste24 than in WT animals (Figure 2B).

As observed previously in WT animals (7), the number of nuclei per millimeter fiber length and average MND size positively and linearly related to their fiber cross-sectional area (CSA) (Figure 2, B and C). However, in Zmpste24-deficient mice, as the number of nuclei per millimeter fiber length did not increase with fiber CSA (Figure 2B), the MND volume was significantly greater than in WT rodents, with larger fibers being more severely affected (Figure 2C). MND measurements provide valuable information about the average volume of cytoplasm controlled by each myonucleus; however, they do not allow characterization of the overall spatial arrangement/organization of myonuclei within the whole fiber. To evaluate this parameter, we started by calculating nearest neighbor (NN) distances using the 3D coordinates of individual nuclei within each single fiber. In WT mice, NN distance was unrelated to CSA and remained constant at ~34 μm (Figure 2D). On the other hand, NN distance was significantly dependent on CSA in Zmpste24-deficient animals (Figure 2D), supporting once again the notion that larger fibers are more severely affected. To further assess the regularity of nuclear positioning within muscle fibers, a distribution or order score (*g*) was calculated as described previously (11). Interestingly, this parameter was not affected by the absence of Zmpste24 (Figure 2E), implying that a regular nuclear spacing is maintained despite differences in nuclear number, MND, and NN distance.

**Figure 3:**
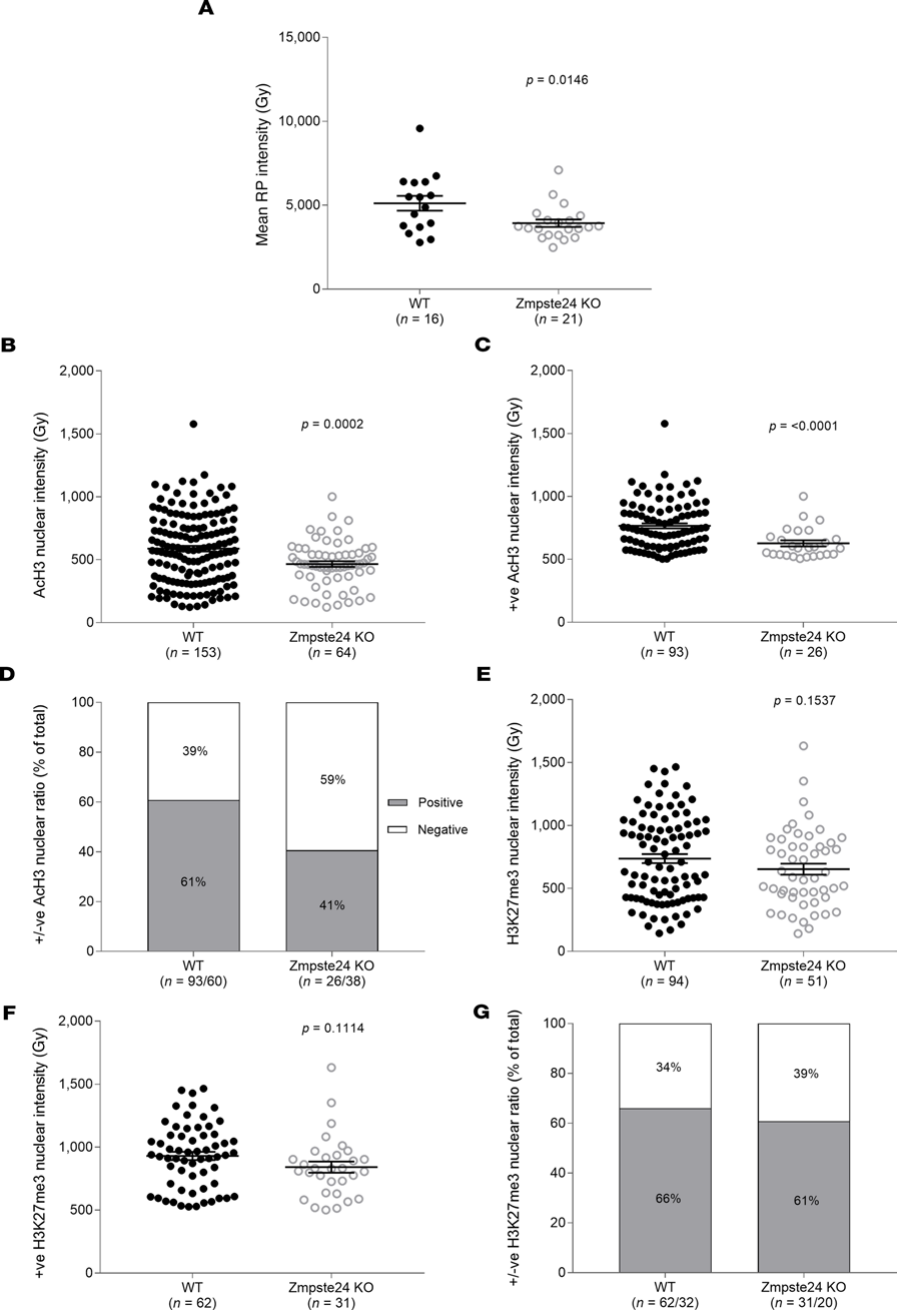
Expression of acetyl-histone H3 (Lys9/Lys14) and actin (phalloidin) in muscle fibers lacking Zmpste24. Mean intensity of (**A**) actin (rhodamine phalloidin [RP]); (**B**–**D**) acetyl-histone H3 (AcH3); (**E**–**G**) H3K27me3 staining in nuclei from WT (*n* = 4, mice, *n* = 65 nuclei) and Zmpste24-deficient (Zmpste24 KO, *n* = 4 mice, *n* = 46 nuclei) muscle fibers. Data are shown as mean ± SD with individual points being single myofibers. Statistical tests included normality tests and *t* tests. +ve, positive; –ve, negative.

**Figure 4:**
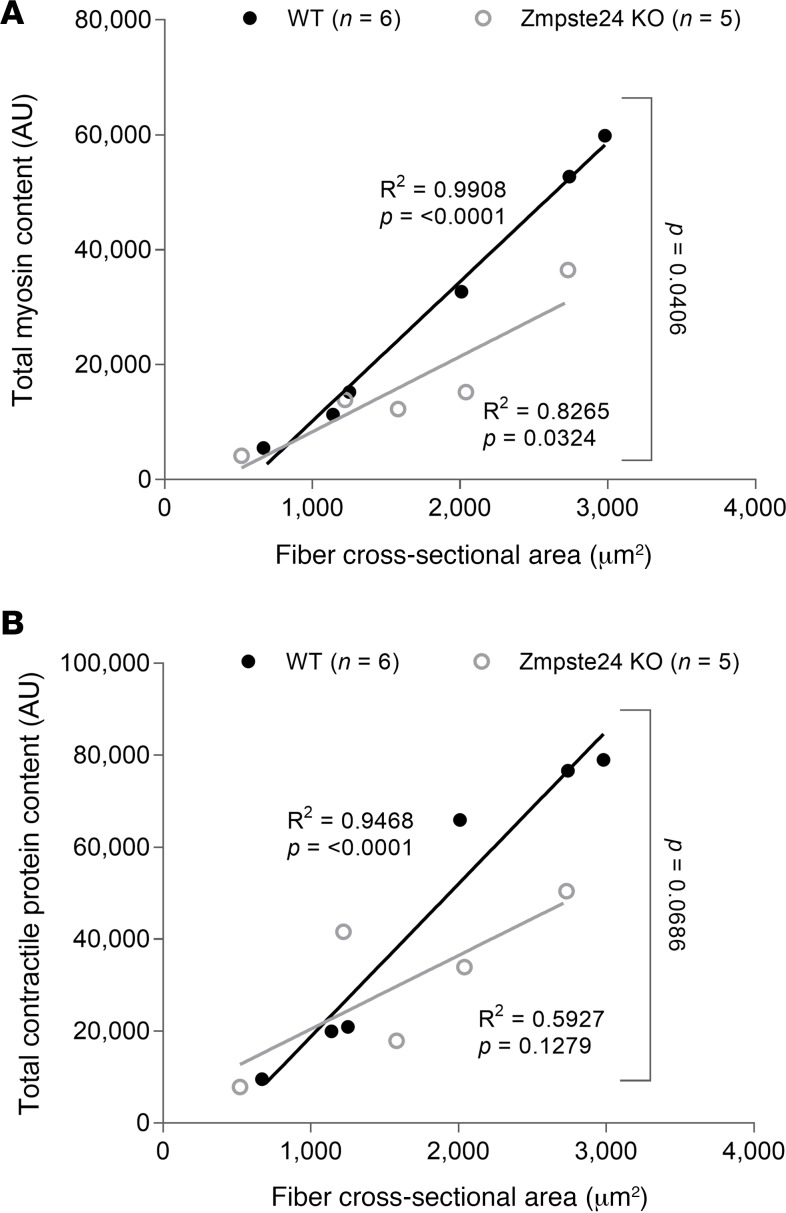
Reduced contractile proteins in the absence of Zmpste24. (**A** and **B**) This figure relates to the TMT6plex experiments. Data are presented as scatterplots, wherein individual points correspond to single muscle fibers (WT, *n* = 3 mice, *n* = 6 fibers; Zmpste24 KO, *n* = 3 mice, *n* = 5 fibers). Statistical tests included normality tests and *t* tests.

Such nuclear abnormalities could be accompanied by modified global gene transcription, since nuclear cooperation is known to be affected by nuclear spacing within muscle fibers (12). To address this possibility, we used acetyl-histone H3 (AcH3) and H3K27me3 as markers of global transcriptional activity. The intensity of AcH3 within individual nuclei was significantly lower in mice lacking Zmpste24 than in WT animals (Figure 3B). Additionally, the mean positive AcH3 intensity (measured by setting a cutoff point for nuclei above 500 Gy of background level) was significantly reduced in Zmpste24-deficient compared with WT counterparts (Figure 3C), resulting in a decreased total positive to negative AcH3 nuclear ratio (Figure 3D). However, no significant differences were observed for H3K27me3 (Figure 3, E–G). Overall, these data suggest potential transcriptional alterations that may affect contractile protein synthesis and content. Indeed, we observed that the mean pixel intensity of rhodamine phalloidin within individual myofibers was lower in the absence of Zmpste24 (Figure 3A). To support our interpretation of these data, we ran a proteomics analysis (TMT6plex experiments) focusing on contractile proteins. The content of myosin and total contractile proteins (including myosin; actin; tropomyosin; troponin I, C, and T) was lower in myofibers from mice lacking Zmpste24 when compared with fibers from WT mice, and this was dependent on fiber size, with a greater effect observed in fibers with larger cross-sectional area (Figure 4).

Together these results suggest that mechanisms related to (i) myonuclear number and (ii) contractile protein content are likely to be involved in the etiology of muscle fiber dysfunction in the presence of prelamin A.

### Muscle fibers lacking Zmpste24 display reduced force production.

To measure the force-generating capacity of myofibers at the contractile level without the confounding effects of possible disrupted sarcoplasmic reticulum Ca^2+^ handling or sarcolemmal excitability, we measured the absolute steady-state isometric force at saturating [Ca^2+^] (pCa 4.50) of 77 membrane-permeabilized fibers. Absolute force was strongly related to CSA, and a positive linear relationship was observed in WT mice (Figure 2F). However, such a relationship was nonexistent in mice lacking Zmpste24 (Figure 2F), demonstrating that larger fibers with disproportionately greater MND sizes (and lower contractile protein content) were more severely impaired (Figure 2, C and F). Specific force, defined as absolute (maximal) force divided by CSA, was significantly lower in mice lacking Zmpste24 than in WT animals (Figure 2G). This force depression can be due to changes in the total number of myosin molecules available, their recruitment upon Ca^2+^ activation and/or their intrinsic cycling and mechanical properties in binding to actin. To distinguish between these potential mechanisms, we started by measuring rigor force (maximum force in the absence of ATP). This parameter was significantly smaller in rodents lacking Zmpste24 than in WT mice (Figure 2H). As rigor force and specific force were depressed to a similar extent (Figure 2G), and as under rigor conditions, all myosin heads are attached because of a very slow dissociation rate (13), we suggest that the Ca^2+^ recruitment of myosin molecules is not affected in the presence of prelamin A. In addition to measuring rigor force, we calculated the rate of force development (*k_tr_*). Zmpste24 deficiency did not have any negative effect on this parameter (38.40 ± 3.00 s^–1^ for mice lacking Zmpste24 versus 39.10 ± 2.50 s^–1^ for WT mice). Taking into consideration that *k_tr_* depends on *f*_app_ + *g_app_*, with *f_app_* being the rate constant for attachment and *g_app_* being the rate constant for detachment (14), the force depression in the presence of prelamin A is not associated with significant changes in myosin cross-bridge cycling properties. Hence, the specific force depression is most likely related to a quantitative reduction in the content of contractile proteins (total number of myosin molecules available) observed in the Zmpste24 mutant mice (Figure 4).

Altogether, our findings unravel, for the first time to our knowledge, a potential link between suboptimal nuclear number and functional contractile efficiency of muscle fibers.

### Reducing the amount of prelamin A in Zmpste24-deficient muscle fibers rescues nuclear arrangement and force-generating capacity.

**Figure 5:**
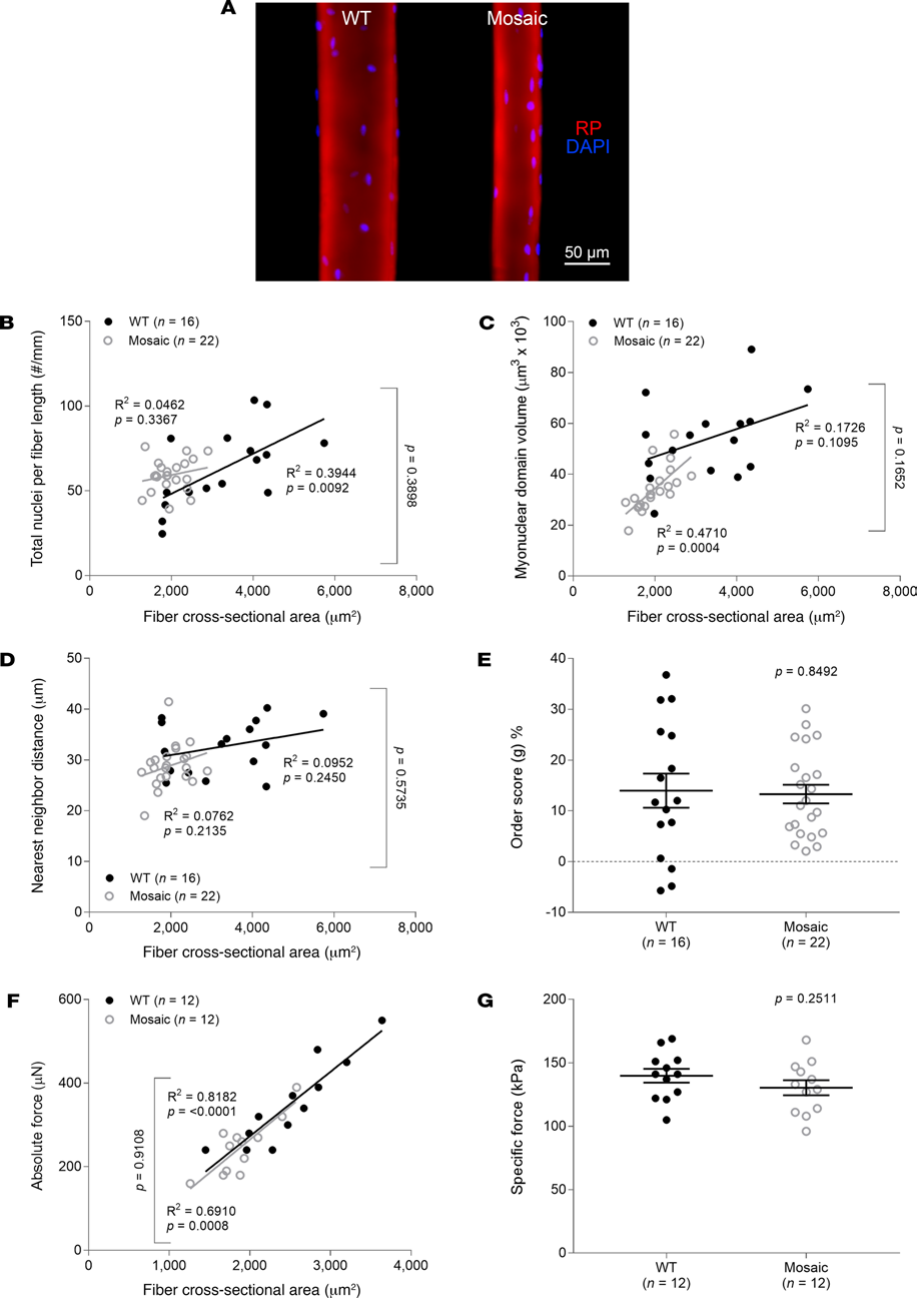
Restoration of myonuclear number and force generation when the number of Zmpste24-deficient nuclei decreases. (**A**) Typical confocal images (×20 objective) of myofibers from 2-month-old WT (*n* = 3 mice, *n* = 16 fibers) and mosaic mice exhibiting similar amounts of Zmpste24-deficient (prelamin A–accumulating) and Zmpste24-proficient (mature lamin A–containing) nuclei in their cells (*n* = 3 mice, *n* = 22 fibers). These fibers were stained for nuclei (DAPI, blue) and actin (rhodamine phalloidin [RP], red). In **B**–**G**, data are presented as mean ± SEM, and as scatter plots wherein individual points correspond to single muscle fibers. Statistical tests included normality tests, *t* tests, and Pearson’s product moment correlation (to evaluate linear relationships).

**Figure 6:**
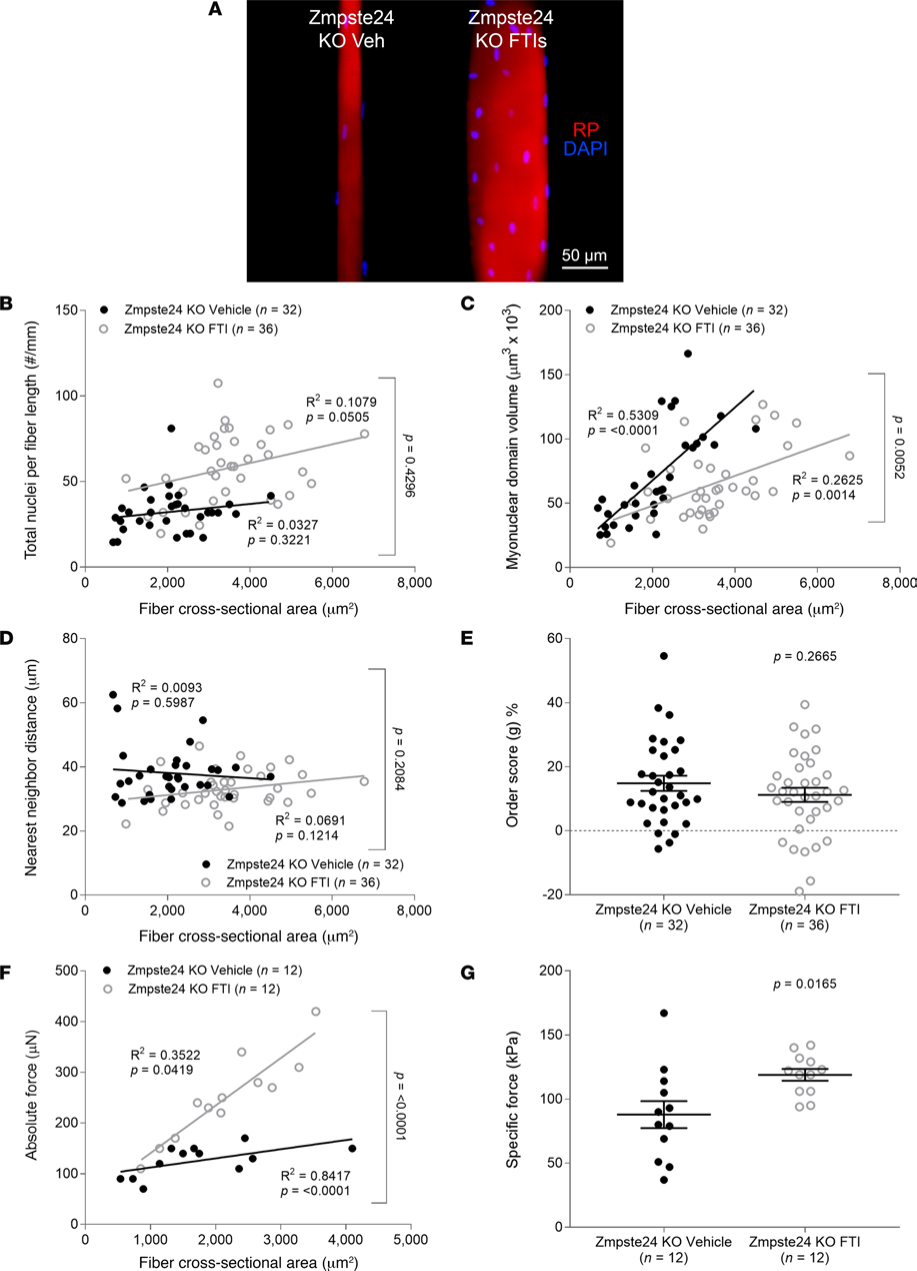
Increases in myonuclear number and force production in response to a farnesyltransferase inhibitor treatment in Zmpste24-deficient mice. (**A**) Typical confocal images (×20 objective) of fibers from 4-month-old mice lacking Zmpste24 treated with a farnesyltransferase inhibitor (FTI, *n* = 2 mice, *n* = 36 fibers) or vehicle (Veh, *n* = 2 mice, *n* = 32 fibers). These fibers were stained for nuclei (DAPI, blue) and actin (rhodamine phalloidin [RP], red). In **B**–**G**, data are presented as mean ± SEM, and as scatter plots wherein individual points correspond to single muscle fibers. Statistical tests included normality tests, *t* tests, and Pearson’s product moment correlation (to evaluate linear relationships).

To support our proposal of a direct correlation between myonuclear number and force production, we used two different approaches. First, we compared fibers from WT animals with those from Zmpste24 mosaic mice known to have equal proportions of Zmpste24-deficient (prelamin A–accumulating) and Zmpste24-proficient (mature lamin A–containing) nuclei in their cells (15). Number of nuclei per millimeter fiber length and specific force were similar in the 2 mouse lines (Figure 5). Second, we evaluated the effects of a farnesyltransferase inhibitor (FTI; ABT-100) on the muscle fiber function of Zmpste24-deficient mice (16). ABT-100 has previously been shown to block the farnesylation of prelamin A, preventing its accumulation at the nuclear envelope level (16). As anticipated, myofibers from FTI-treated rodents exhibited rescued number of nuclei, MND sizes, and force-generating capacity when compared with vehicle-treated animals (Figure 6).

### Lamin A–deficient muscle fibers have subtle alterations in nuclear number but a normal ability to generate force.

**Figure 7:**
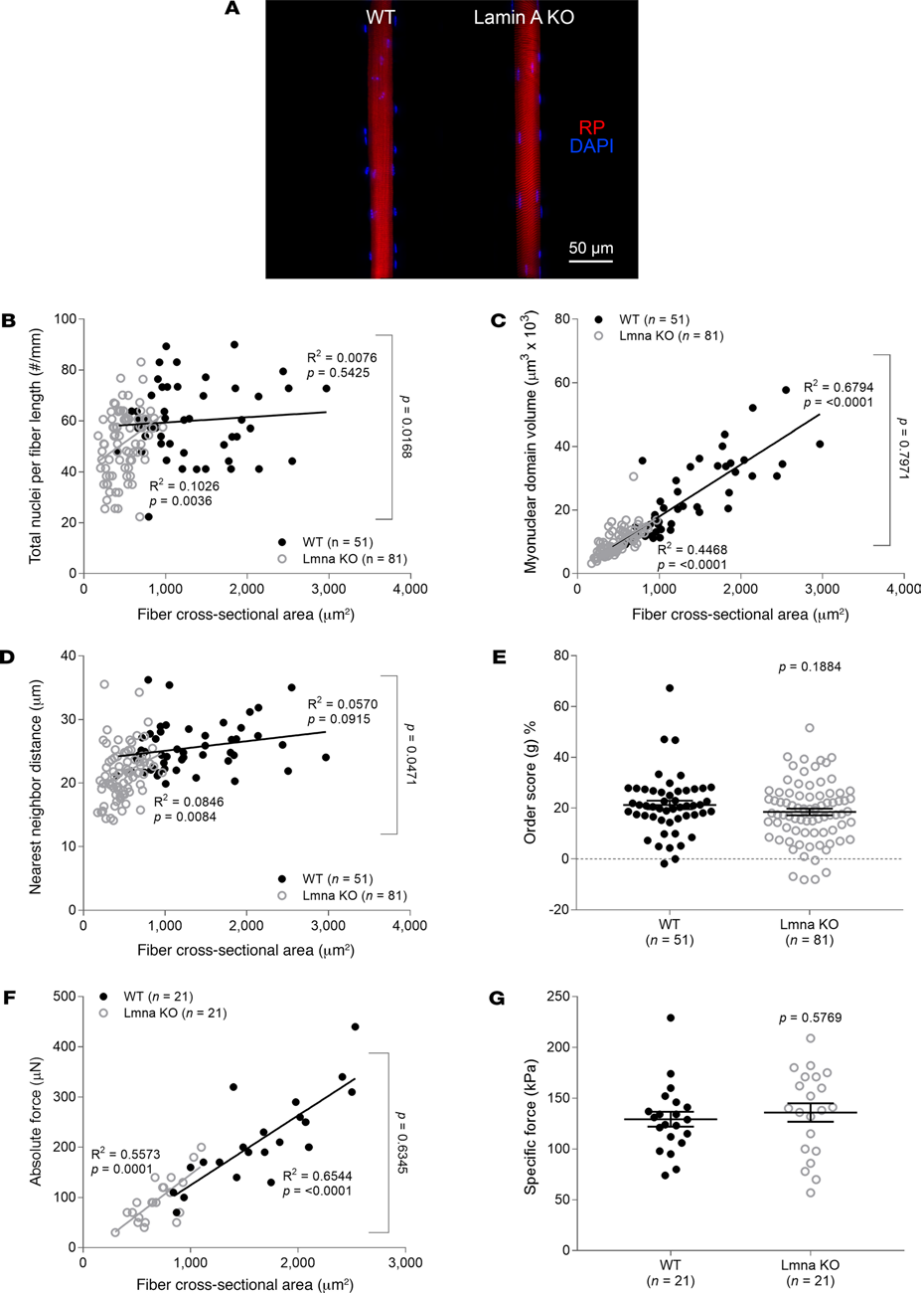
Disrupted myonuclear spatial arrangement and contractility in absence of lamin A. (**A**) Typical confocal images (×20 objective) of muscle fibers from typical 5-week-old WT (*n* = 4 mice, *n* = 51 fibers) and homozygous lamin A–deficient (Lmna KO, *n* = 4 mice, *n* = 81 fibers) mice. These fibers were stained for nuclei (DAPI, blue) and actin (rhodamine phalloidin [RP], red). In **B**–**G**, data are presented as mean ± SEM, and as scatter plots wherein individual points correspond to single muscle fibers. Statistical tests included normality tests, *t* tests, and Pearson’s product moment correlation (to evaluate linear relationships).

**Figure 8:**
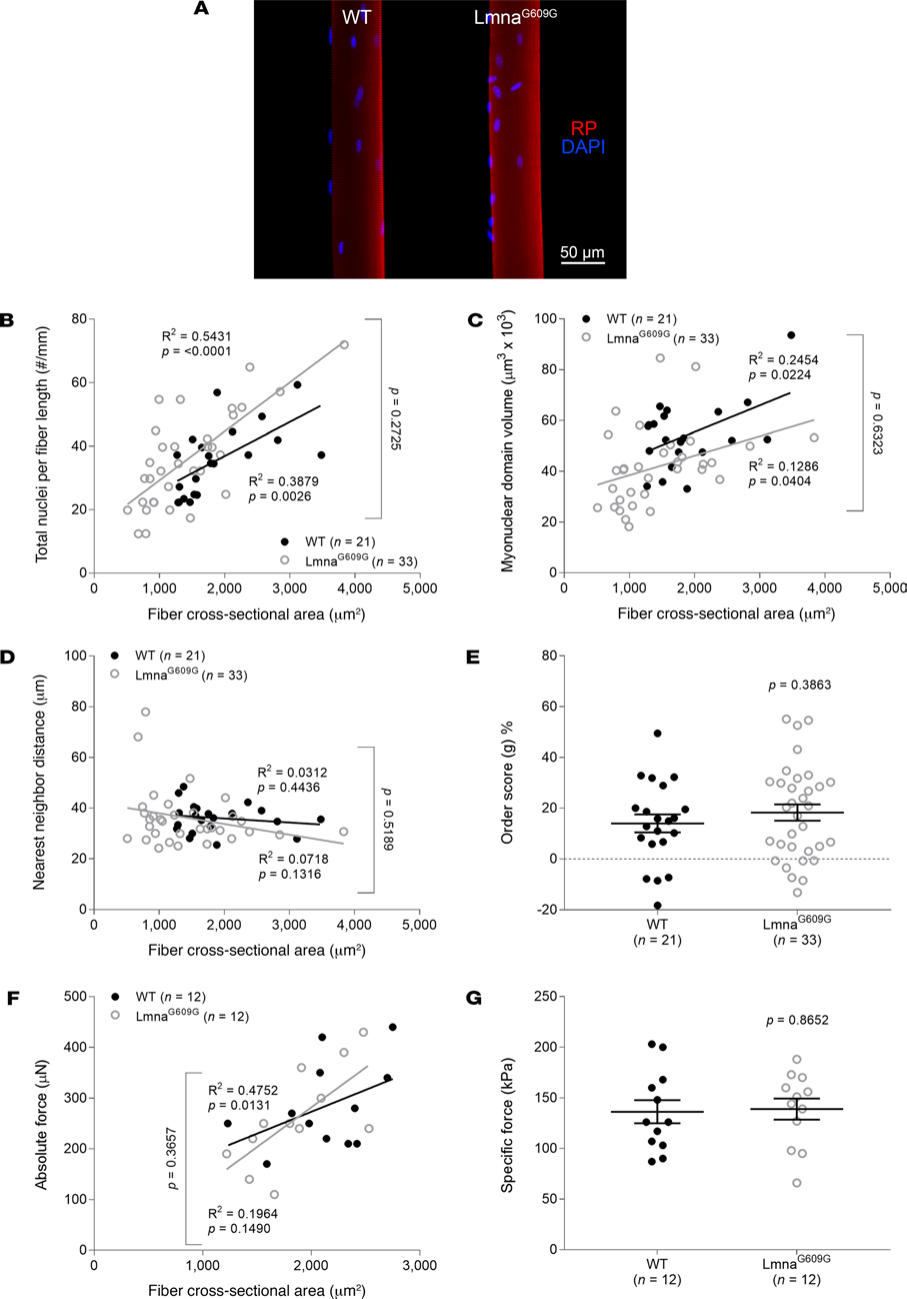
Normal myonuclear arrangement and contractility in the presence of a truncated form of lamin A, progerin. (**A**) Typical confocal images (×20 objective) of muscle fibers from typical 12-week-old WT (*n* = 4 mice, *n* = 21 fibers) and homozygous lamin A–deficient (Lmna G609G/G609G, *n* = 4 mice, *n* = 33 fibers) mice. These fibers were stained for nuclei (DAPI, blue) and actin (rhodamine phalloidin [RP], red). In **B**–**G**, data are presented as mean ± SEM, and as scatter plots wherein individual points correspond to single muscle fibers. Statistical tests included normality tests, *t* tests, and Pearson’s product moment correlation (to evaluate linear relationships).

Prior work from our laboratory and by others has shown that Zmpste24-deficient mice display an accumulation of prelamin A and a deficiency of lamin A (8, 9). Thus, the above results may originate from either of these two alterations or from both. To further investigate the effects of a specific lamin A deficiency, without the confounding effects of high levels of prelamin A, we studied a transgenic mouse model lacking A-type lamins (lamin A and C) (17, 18) (Figure 7A). Even though myofibers from lamin A–deficient mice had a significantly lower number of nuclei per millimeter fiber length than those from WT animals (Figure 7B), the regression lines of the MND size–CSA and absolute force–CSA relationships were normal (Figure 7, C and F). Additionally, the order score (*g*) and intrinsic force-generating capacity did not differ between the genotypes (Figure 7, D, E, and G). Similar results were observed for mice accumulating progerin, which is a truncated form of lamin A (Figure 8).

Taken together these results suggest that the observed defects in Zmpste24-deficient mice resulted primarily from an accumulation of prelamin A rather than a lack of lamin A/C and, strikingly, that the defects are not phenocopied by expression of progerin, which like prelamin A retains a C-terminal CAAX domain that alters a number of other functional parameters of the intermediate filament protein.

### Disruption of nesprin-1, but not SUN1, leads to nuclear and force alterations similar to those in muscle fibers lacking Zmpste24.

**Figure 9:**
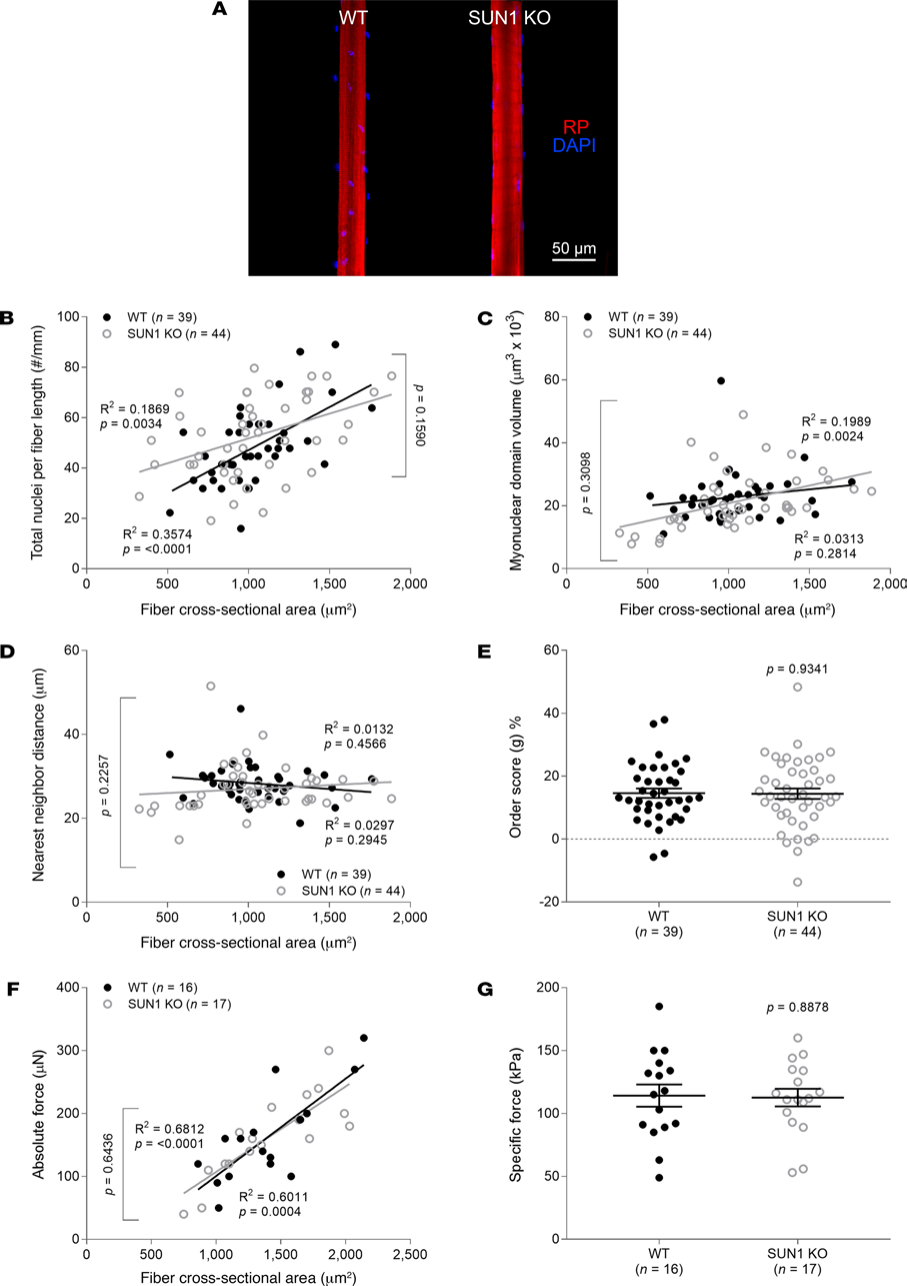
Normal nuclear organization and force production in SUN1-deficient muscle fibers. (**A**) Typical confocal images (×20 objective) of myofibers from 4-month-old WT (*n* = 4 mice, *n* = 39 fibers) and homozygous SUN1-deficient (SUN1 KO, *n* = 4 mice, *n* = 44 fibers) mice. These fibers were stained for nuclei (DAPI, blue) and actin (rhodamine phalloidin [RP], red). In **B**–**G**, data are presented as mean ± SEM, and as scatter plots wherein individual points correspond to single muscle fibers. Statistical tests included normality tests, *t* tests, and Pearson’s product moment correlation (to evaluate linear relationships).

**Figure 10:**
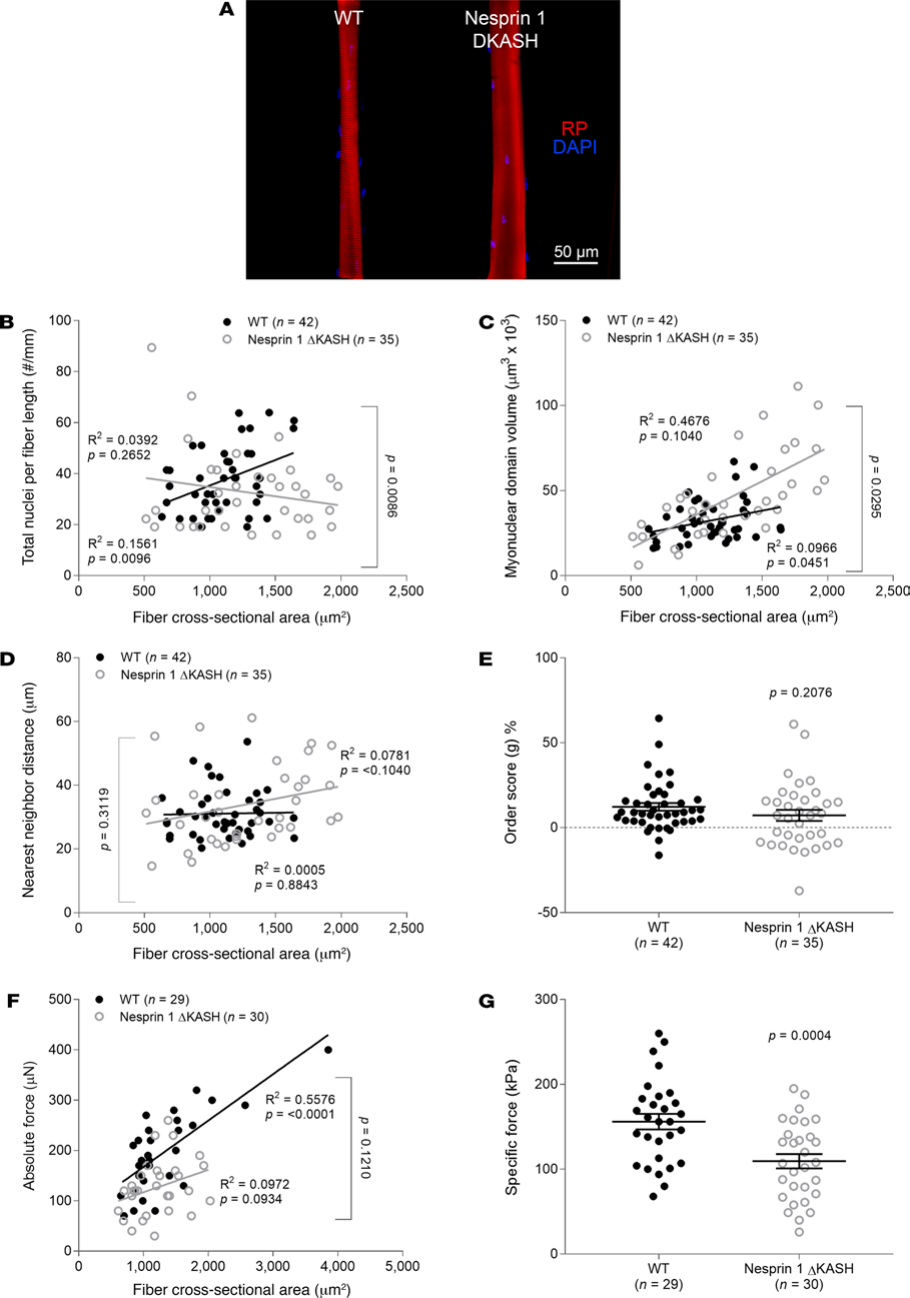
Changes in nuclear positioning and force generation when the KASH domain of nesprin-1 is lacking. (**A**) Typical confocal images (×20 objective) of myofibers from 3-month old WT (*n* = 4 mice, *n* = 42 fibers) and homozygous KASH domain of nesprin-1–deficient (Nesprin-1 ΔKASH, *n* = 4 mice, *n* = 35 fibers) mice. These fibers were stained for nuclei (DAPI, blue) and actin (rhodamine phalloidin [RP], red). In **B**–**G**, data are presented as mean ± SEM, and as scatter plots wherein individual points correspond to single muscle fibers. Statistical tests included normality tests, *t* tests, and Pearson’s product moment correlation (to evaluate linear relationships).

Since properties of nuclei are known to be dependent on their membrane (19–22), we also examined mice with alterations in other linker of nucleoskeleton and cytoskeleton (LINC) complex proteins, in particular SUN1 and nesprin-1 (located within the nuclear envelope). Mice lacking SUN1 closely mirrored WT rodents (Figure 9). In contrast, mice lacking the KASH domain of nesprin-1 displayed changes similar to those in Zmpste24-deficient animals (Figure 10). In fact, we observed aberrant nuclear number–CSA (Figure 10B), MND size–CSA (Figure 10C), NN distance–CSA (Figure 10D), and absolute force–CSA relationships (Figure 10F), as well as reductions in specific force (Figure 10G).

These findings demonstrate variabilities between LINC complex proteins in their determination of nuclear number, MND size, and cellular force generation. Future studies are now warranted to decipher the exact mechanisms by which various nuclear envelope proteins specifically regulate (or not) the abundance of myonuclei.

## Discussion

In the present work, we aimed to unravel the mechanisms by which prelamin A accumulation modifies muscle fiber function.

### Suboptimal MND sizes.

In the presence of prelamin A, we observed a lower number of nuclei along the length of myofibers, together with an increase in NN distances and MND volumes. This decrease in nuclear number might be related to a change in nuclear turnover, whereby myonuclei are not incorporated as efficiently, or are extensively lost. Previous studies using the same mouse model lacking Zmpste24 have observed a defect in the process of nuclear incorporation into myofibers, due to a reduction in the total muscle stem cell (satellite cell) pool, as well as lower rates of proliferation and differentiation (23, 24). Overall, satellite cell activation and myoblast fusion with muscle fibers have been shown to be impaired when the nuclear envelope is altered (24–26), suggesting that a change in the rate of nuclear incorporation might be the primary cause of the low number of nuclei in muscle fibers where prelamin A accumulates. Insufficient nuclear number may also be a mechanism whereby the myofibers of Zmpste24-deficient mice cannot sustain fiber sizes as large as those of WT mice. This hypothesis is supported by our proteomics data, which show reduced contractile protein content in the myofibers of Zmpste24-deficient mice, particularly in larger fibers.

The maintenance of nuclear number, NN distance, and MND size is a prerequisite for preserving the intrinsic force-generating capacity of muscle fibers during pathophysiological conditions (27–29). Hence, the decrease in the number of nuclei and concomitant increase in NN distance and MND volume in the presence of prelamin A may explain the loss in specific force in membrane-permeabilized fibers (where neuromuscular junction, excitation-contraction coupling, and sarcoplasmic reticulum Ca^2+^ release are not involved). This is in accordance with a recent study investigating myonuclear organization and force production in transgenic mice lacking myostatin, which suggested that abnormally hypertrophied fibers were limited in their force production through the inability to maintain optimal MND sizes (29). The absence of myostatin or Zmpste24 may then share similar cascades of suboptimal or negative cellular events. A lower incorporation and/or increased loss of myonuclei may require the remaining myonuclei to extend their individual influence to unusually large cytoplasmic volumes beyond their maximum functional capacity (physiological “ceiling”), preventing optimal distribution of gene products and protein synthesis, and ultimately leading to a suboptimal density of contractile proteins (i.e., myosin). Therefore, intrinsic myofiber force production is decreased, and this results in skeletal muscle weakness. Further supporting this interpretation, our results clearly show that specific force is preferentially reduced in larger myofibers with larger MND sizes and aberrant NN distances, and that this is concurrent with reduced contractile protein content in larger fibers. Even though we believe that these phenomena are crucial, other unknown factors may also contribute to defects in muscles expressing prelamin A.

### Preserved nuclear distribution.

As mentioned earlier, myonuclei are well known to be evenly spaced along the surface of muscle fibers. Such distribution is crucial to facilitate lower cytosolic transport distances (11) and to favor a better control of chromosomal architecture and transcriptional regulation (30). Interestingly, we found that the overall distribution of nuclei was preserved in all models studied, implying that proper inter-nuclear communication is maintained (31).

### What causes nuclear dysfunction?

Our data on mice lacking lamin A/C tend to demonstrate that a deficiency in this protein has different consequences for myonuclear behavior than Zmpste24 deficiency. This confirms our initial hypothesis that the presence of prelamin A, rather than the absence of lamin A/C, is the major determinant underlying the above changes in NN distance and MND size. In addition, mice with an accumulation of progerin did not show significant defects, again suggesting that the effects are specific to the presence of prelamin A, rather than other processed forms of lamin A.

It is worth noting that mice lacking A-type lamins develop a muscular dystrophy phenotype, consistent with a wide range of human *LMNA* mutations associated with this disease. Therefore, while both mice expressing prelamin A and those lacking A-type lamins have muscle defects, our findings indicate that the etiology of muscle dysfunction may be different. In the absence of A-type lamins, increased degeneration of both myofibers and myonuclei has been observed (32, 33), in addition to differentiation defects (26), suggesting a combination of factors distinct to those associated with expression of prelamin A contribute to dysfunction. Further close comparison of different mouse models with lamin A/C mutations will be required to delineate these differences.

### Conclusion.

Prelamin A dysregulates myonuclear abundance, NN distance, MND size, and intrinsic force-generating capacity. This mechanism may contribute to muscle fiber weakness in premature aging syndromes related to prelamin A accumulation.

## Methods

### Animals.

Various transgenic mouse models were used in the present study, i.e., Zmpste24-KO (9), Zmpste24^–/–^ Hprt^Z24/+^ (referred to herein as mosaic) (15), lamin A–KO (34), lamin A^G609G/G609G^ (referred to herein as lamin A^G609G^) (34, 35), SUN1-KO (36) mice, and mice with an ablation in the KASH domain of nesprin-1, which disrupts the insertion of this protein into the nuclear membrane (referred to herein as nesprin-1 ΔKASH) (37). Corresponding age-matched WT siblings were also sacrificed, and fast-contracting extensor digitorum longus (EDL) skeletal muscles were dissected.

### FTI.

ABT-100 was mixed in drinking water containing 0.4% hydroxy methyl propyl cellulose and 1.0% ethanol at a concentration of 0.3 mg/ml, so as to deliver a dose of 39 mg/kg/d. The vehicle control consisted of drinking water with 0.4% hydroxy methyl propyl cellulose and 1.0% ethanol. Mice drank the vehicle or ABT-100 for 15 weeks starting at 5 weeks of age before euthanization and muscle dissection (16).

### Muscle fiber permeabilization.

Muscle samples were placed in relaxing solution at 4°C. Bundles of approximately 50 myofibers were dissected free and then tied with surgical silk to glass capillary tubes at slightly stretched lengths. They were then treated with skinning solution (relaxing solution containing glycerol; 50:50 v/v) for 24 hours at 4°C, after which they were transferred to –20°C. For long-term storage the muscle bundles were treated with sucrose, a cryoprotectant, within 1–2 weeks (38), detached from the capillary tubes, snap frozen in liquid nitrogen-chilled propane, and stored at –80°C.

### Single myofiber force production.

On the day of experiment, bundles were de-sucrosed and transferred to a relaxing solution, and single myofibers were dissected. They were then individually attached between connectors leading to a force transducer (model 400A; Aurora Scientific) and a lever arm system (model 308B; Aurora Scientific). Sarcomere length was set to ≈2.50 μm and the temperature to 15°C (39–41). Fiber CSA was estimated from the width and depth, assuming an elliptical circumference. The absolute maximal isometric force generation was calculated as the difference between the total tension in the activating solution (pCa 4.50) and the resting tension measured in the same myofiber while in the relaxing solution (pCa 9.0). Specific force was defined as absolute force divided by CSA. Note that myofibers included in the analysis (i) were able to sustain 3 consecutive maximum activations without any force depression >10%; and (ii) had preserved sarcomere structures after the 3 maximum activations.

**Figure 11:**
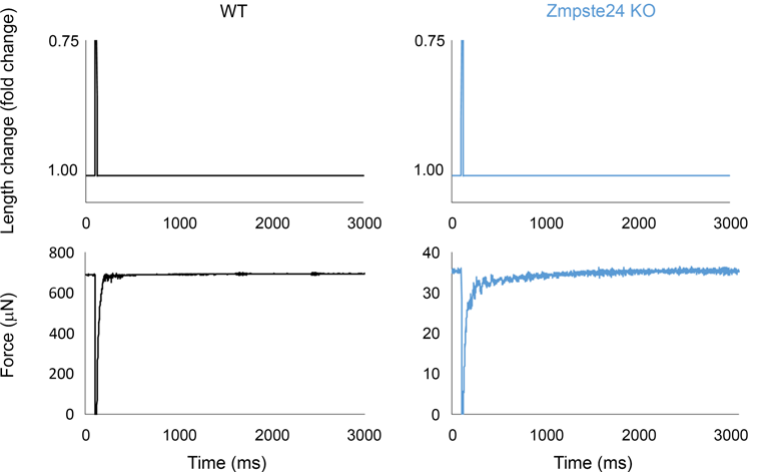
Measurement of the rate of force redevelopment. Original recordings for the calculation of the rate of force redevelopment. Length and force signals of myofibers from one 4-month-old WT animal and from one age-matched homozygous Zmpste24-deficient (Zmpste24 KO) mouse are shown. Note that the time to half force recovery was 16 ms for WT and 18 ms for Zmpste24 KO. Hence, the rate of force redevelopment was 43.30 s^–1^ for WT and 38.50 s^–1^ for Zmpste24 KO. Note also that the force recovery is close to 100% for these original recordings as well as for most of all the other myofibers.

Measurement of the apparent rate constants of force redevelopment (*k_tr_*) involved a mechanical slack-restretch maneuver. Each fiber was transferred from the relaxing to activating solution and allowed to generate steady-state force. The fiber was then rapidly slackened (within 1–2 ms) by 25% of its original length, resulting in a rapid reduction in force to near zero. This was followed by a brief period of unloaded shortening (20 ms), after which the preparation was rapidly restretched to its original length (13). *k_tr_* was estimated by linear transformation of the half-time of force redevelopment (*k_tr_* = 0.693/*t_1/2_*) as described previously (13) (Figure 11).

### Solutions.

Relaxing and activating solutions contained 4 mM Mg-ATP, 1 mM free Mg^2+^, 20 mM imidazole, 7 mM EGTA, 14.5 mM creatine phosphate, and KCl to adjust the ionic strength to 180 mM and pH to 7.0. The concentrations of free Ca^2+^ were 10^–9.00^ M (relaxing solution) and 10^–4.50^ M (activating solution).

### Nuclear organization of single fibers.

Single muscle fibers were dissected following the same procedure as above. Arrays of approximately 9 myofibers were prepared at room temperature (RT). For each myofiber, both ends were clamped to half-split copper meshes designed for electron microscopy (SPI G100 2010C-XA, width 3 mm), which had been glued to cover slips (Menzel-Gläser, 22 × 50 mm, thickness 0.13–0.16 mm). For measurement of nuclear coordinates, fibers were mounted at a fixed sarcomere length of ≈2.20 μm. This was a prerequisite for exact determination of spatial organization of nuclei, as it allowed accurate comparisons between myofibers (7, 29, 42).

At RT, arrays were fixed in 4% PFA for 10 minutes, washed 3 times in PBS, further permeabilized in 0.1% Triton-X100/PBS for 10 minutes, and subsequently subjected to actin staining (rhodamine-conjugated phalloidin at 1:100 in PBS, Molecular Probes, R415) and nuclear staining (DAPI at 1:1,000 in PBS, Molecular Probes, D3571). Images were acquired using a confocal microscope (Zeiss Axiovert 200, objectives ×20, ×40, and ×100) equipped with a CARV II confocal imager (BD Biosciences). To visualize muscle fibers in 3D, stacks of 100 images were acquired (1 μm *Z* increments) and analyzed with a custom-made MATLAB (MathWorks) program. Membrane-permeabilized fibers did not display any Pax7-positive satellite cells. It should be noted that, in order to measure how ordered the nuclear distribution for a particular fiber was, the individual NN distances were calculated for the experimental data. Then, a theoretical optimal and a theoretical random distribution was simulated, based on fiber dimensions and nuclear number. We denote the experimental, random, and optimal means by *M_E_*, *M_R_* and *M_O_*, respectively. An order-score, *g* (11), was then calculated as: *g* = (*M_E_* – *M_R_*)/(*M_O_* – *M_R_*).

### Fluorescence labeling.

Fibers were PFA-fixed and Triton-permeabilized as described above (*Nuclear organization of single fibers*). Fibers were treated with mouse-on-mouse block (Vector Laboratories, BMK-2202) for 1 hour, blocked in 10% goat serum in PBS (Sigma-Aldrich, G9023) for 30 minutes, and treated with primary antibodies diluted in goat serum blocking buffer. Prelamin A was visualized using as primary antibody Santa Cruz Biotechnology Inc. sc-6214, c-20 (secondary antibody: Alexa Fluor 488, Invitrogen, A-11078) and Pax7 using Developmental Studies Hybridoma Bank (DSHB) AB 528428 as primary antibody (secondary antibody: Alexa Fluor 488, Invitrogen, A-11001). Lamin A (Abcam, ab8980) and nesprin-1 (Abcam, ab192234) were matched with Alexa Fluor 488 and 594, respectively (Invitrogen, A-11001 and A-11012). Acetyl-histone H3 (Lys9/Lys14) antibody (Cell Signaling Technology, 9677) matched with Alexa Fluor 594, respectively (Invitrogen, A-11012).

### LC-MS/MS identification and quantitative analysis of protein.

For preparation, 3-mm-long muscle fibers were dissected and their CSAs calculated as above. These fibers were then placed in tubes containing 25 μl Tris-Triton lysis buffer (10mM Tris [pH 7.4], 100 mM NaCl, 1 mM EDTA, 1 mM EGTA, 1% Triton X-100, 10% glycerol, 0.1% SDS, 0.5% deoxycholate). Prior to enzymatic digestion and labeling, the samples were loaded into a stack gel for lysis buffer cleanup to eliminate chemical interference at the labeling stage and to compress the whole proteome into a single band. Sample volumes were dried by half in a SpeedVac (Thermo Fisher Scientific) with the volume replaced by Laemmli buffer (2×) and boiled for 10 minutes at 96°C. Reduced samples were loaded onto a 10% Bis-Tris NuPAGE gel and resolved for 10 minutes (100 V; 59 mA; 6 W) to “stack” the whole sample into a single band. Protein bands were visualized using Imperial protein stain (Thermo Fisher Scientific).

For digestion and peptide labeling with tandem mass tag (TMT), in-gel reduction, alkylation, and digestion with trypsin were performed on all the samples prior to subsequent isobaric mass tag labeling (43). Each sample was treated individually with labels added at a 1:1 ratio.

For LC-MS/MS tandem mass spectrometry, the combined TMT-labeled peptide samples were resuspended in a solution containing water:acetonitrile:trifluoroacetic acid (98%:2%:0.05%) and analyzed by LC-MS/MS. Chromatographic separations were performed using an Ultimate 3000 UHPLC system (Thermo Fisher Scientific). A 10-μl injection of peptides was resolved by reversed phase chromatography on a 75-μm C18 column (50 cm) using a 3-step linear gradient of acetonitrile in 0.1% formic acid. The gradient was delivered to elute the peptides at a flow rate of 250 nl/min over 120 minutes. The eluate was ionized by electrospray ionization using an Orbitrap Fusion Lumos (Thermo Fisher Scientific) operating under Xcalibur v4.1. The instrument was programmed to acquire in automated data-dependent switching mode. The selection of precursor ions was based on their intensity for sequencing by higher-energy C-trap dissociation (HCD) for peptide identification and reporter ion fragmentation. Selection of precursor ions was also based on their intensity for sequencing by HCD in a TopN method. The MS/MS analyses were conducted using higher than normal collision energy profiles that were chosen based on the mass-to-charge ratio (*m/z*) and the charge state of the peptide. To increase fragmented peptide coverage and reporter ion intensities, a further synchronous precursor scan (SPS) of the top 5 most intense peaks using MS3 (Thermo Fisher Scientific) was performed.

### Database searching.

Raw mass spectrometry data were processed into peak list files using Proteome Discoverer (Thermo Fisher Scientific; v2.2). The raw data file was processed and searched using the Mascot search algorithm (v2.6.0; Matrix Science) and the SEQUEST search algorithm (44) against the current mouse database curated within UniProt (https://www.uniprot.org/).

### Bioinformatics.

Following processing with Proteome Discoverer, the result file was exported into Perseus (v1.6.3; http://www.perseus-framework.org) for qualitative and quantitative data analysis.

### Statistics.

Data are presented as mean ± SEM. Statistical analysis was performed using SPSS Statistics 23 software (IBM) and included normality tests, as well as *t* tests, ANOVAs, and Pearson’s product moment correlation (to evaluate linear relationships). Statistical significance was set to *P* < 0.05.

### Study approval.

The Institutional Animal Care and Use Committees of the University of Minnesota, University of Oviedo, University of California Los Angeles, Buck Institute for Research on Aging, University of Würzburg, and Northwestern University approved all animal procedures.

## Author contributions

MJP, EMM, LGF, CLO, BKK, DAL, and JO contributed to the conception and design of the work. YL, JAR, MN, VAS, SL, CYL, YMH, MJP, MA, SDRH, SGY, LGF, YE, CLO, BKK, DAL, and JO did the acquisition, analysis, and interpretation of data. YL, JAR, MN, VAS, SL, CYL, YMH, MJP, MA, SDRH, SGY, LGF, YE, CLO, BKK, DAL, and JO drafted the work and revised it critically. YL, JAR, MN, VAS, SL, CYL, YMH, MJP, MA, SDRH, SGY, LGF, YE, CLO, BKK, DAL, and JO approved the final version of the manuscript to be published. YL, JAR, MN, VAS, SL, CYL, YMH, MJP, MA, EMM, SDRH, SGY, LGF, YE, CLO, BKK, DAL, and JO agreed on all aspects of the work.

## Version 1 10/04/2018

Electronic publication
